# The Contribution of Microglia to the Development and Maturation of the Visual System

**DOI:** 10.3389/fncel.2021.659843

**Published:** 2021-04-23

**Authors:** Michael A. Dixon, Ursula Greferath, Erica L. Fletcher, Andrew I. Jobling

**Affiliations:** Department of Anatomy and Physiology, The University of Melbourne, Melbourne, VIC, Australia

**Keywords:** microglia, neural maturation, plasticity, neurogenesis, mononuclear phagocyte

## Abstract

Microglia, the resident immune cells of the central nervous system (CNS), were once considered quiescent cells that sat in readiness for reacting to disease and injury. Over the last decade, however, it has become clear that microglia play essential roles in maintaining the normal nervous system. The retina is an easily accessible part of the central nervous system and therefore much has been learned about the function of microglia from studies in the retina and visual system. Anatomically, microglia have processes that contact all synapses within the retina, as well as blood vessels in the major vascular plexuses. Microglia contribute to development of the visual system by contributing to neurogenesis, maturation of cone photoreceptors, as well as refining synaptic contacts. They can respond to neural signals and in turn release a range of cytokines and neurotrophic factors that have downstream consequences on neural function. Moreover, in light of their extensive contact with blood vessels, they are also essential for regulation of vascular development and integrity. This review article summarizes what we have learned about the role of microglia in maintaining the normal visual system and how this has helped in understanding their role in the central nervous system more broadly.

## Introduction

Microglia, the resident immune cells of the central nervous system (CNS), have emerged as key cells contributing to the development and maturation of the CNS, as well as having roles in homeostasis of the adult nervous system (Rathnasamy et al., 2019). While microglia were once thought to be largely quiescent, only responding to damage or disease, it is now known that microglia dynamically survey the parenchyma and play critical roles in maintaining normal neural function (Nimmerjahn et al., 2005; Jobling et al., 2018b). A great deal has been learned about the roles of microglia in neural homeostasis from studies in the visual system because it is a tractable system and an easily accessible part of the CNS.

**Figure 1:**
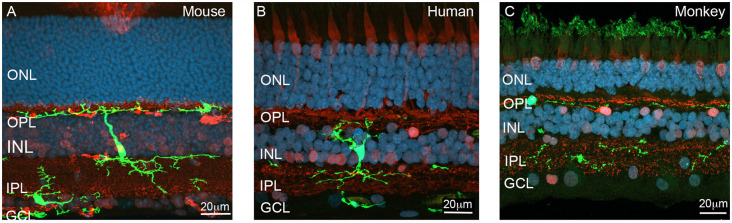
Localization of microglia across the various layers of the retina. **(A)** Vertical section of the adult Cx3CR1^+/GFP^ mouse retina showing a reporter labeled microglial cell (green, EGFP) extending processes from the outer retina (OPL) to the inner retina (IPL). The section was also immunolabeled for the synapse (ribbon) marker, ribeye (red), while nuclei were visualized with bisbenzimide (blue). A second microglia cell (green) is located within the (GCL). **(B)** Vertical section of the human immunolabeled for the microglial marker, IbA1 (green) and calretinin (red). Nuclei are labeled with bisbenzimide (blue). The microglial cell is a large cell that extends processes into both the inner and outer retina where is contacts synapses. **(C)** Vertical section of monkey retina immunolabeled for microglia (IbA1, green) and a combination of the synapse marker, ribeye and the neuronal marker calbindin (red). Abbreviations: ONL, outer nuclear layer; OPL, outer plexiform layer; INL, inner nuclear layer; IPL, inner plexiform layer; GCL, ganglion cell layer. Scale 20 μm.

Visual perception depends on the formation and maturation of complex neural circuits within the retina, as well as a number of higher brain regions. As shown in Figure 1, microglia are localized in the normal retina within three main regions—including the outer plexiform layer (OPL), a synaptic layer consisting of the synapses between photoreceptors and second order neurons (bipolar cells and horizontal cells); the inner plexiform layer (IPL) where the second order neurons, bipolar cells, form synapses with ganglion cells and amacrine cells; and the nerve fiber layer (NFL), where axons of the output neurons of the retina, retinal ganglion cells, are located. Microglia have processes that extend to contact synapses within the retina, including photoreceptor terminals and synapses within the inner retina of both rodents and humans (Figure 1; Wang et al., 2016; Singaravelu et al., 2017; Jobling et al., 2018b). Microglial association with blood vessels is also evident in the OPL and NFL, where they have processes that wrap around capillaries. There are a range of brain regions that are the target of different classes of retinal ganglion cells, the most important of which are the lateral geniculate nucleus (LGN), suprachiasmatic nucleus of the hypothalamus, and optical pretectal nucleus within the midbrain. During development, targeting of ganglion cell axons to these regions, as well as the refinement of synaptic contacts within each of these brain regions, is critical for formation of functional neural circuits (Stevens et al., 2007). This neural refinement is dependent on a visually driven process that is critical for visual perception. Recent evidence suggests that microglia play a critical role in this process.

**Figure 2:**
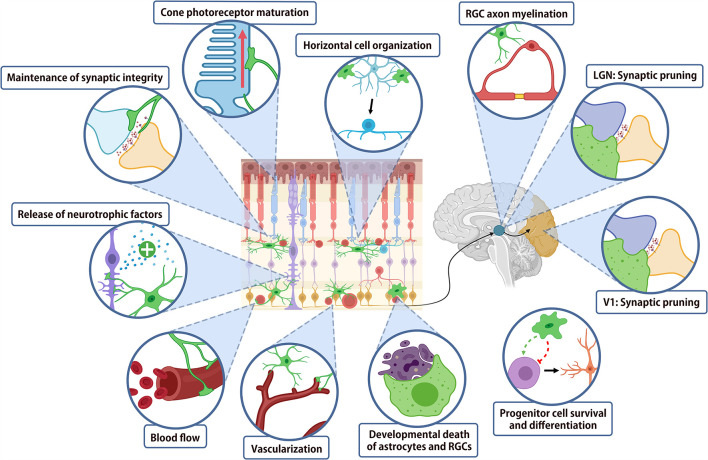
Summary of microglial function in retina and higher order visual centers. Schematic diagram summarizing the major functions of microglia in the development and homeostasis of the visual system.

While the majority of work investigating the role of microglia in normal CNS architecture has been performed in the brain, the readily accessible retina provides a useful model system in order to investigate these changes. A summary of the roles of microglia in the normal nervous system is shown in Figure 2. During development, microglia play critical roles in virtually all stages of neural maturation, from regulating the neural progenitor cell populations, neural maturation and synaptic refinement and plasticity. Microglia also contribute to CNS development by mediating astrocyte growth, regulating myelinogenesis, oligodendrocyte progenitor cell growth and differentiation, whilst also playing a role in blood vessel development. In the mature CNS, microglia express receptors for numerous neurotransmitters, allowing them to continuously monitor and respond to neuronal activity (Wake et al., 2009; Fontainhas et al., 2011). This activity-dependent modulation of neuronal signaling by microglia is important for regulating neural plasticity. Finally, microglia may contribute to regulating the vasculature and blood-retinal or blood-brain barrier.

Underpinning the diverse functions that microglia play during development, maturation and in the adult nervous system is a highly complex and heterogenous transcriptome. Indeed, recent transcriptomic studies using single cell RNA sequencing on populations of microglia isolated at different ages from embryonic day 14.5 to adult shows considerable diversity in expression during early development, with less heterogeneity observed in adult microglia (Hammond et al., 2019; Li et al., 2019). A comparison of microglia isolated from mouse and human brains demonstrates that microglia can be segregated (clustered) based on their transcriptome, with some differences in gene expression noted in each (Masuda et al., 2019). Differences in morphology, density and potentially local environment in different regions of the brain is also associated with variations in microglial transcriptome in both the mouse (Grabert et al., 2016) and human brain (Bottcher et al., 2019). Less is known about the variation in transcriptome across subclasses (or clusters) of microglia isolated from the normal retina, although it has been recently shown that there are at least two different types of microglia in the normal retina—those in the inner retina that are functionally dependent on IL34 and those isolated located in the outer retina that show IL34 independent functions (O’Koren et al., 2019).

While a significant amount of work has been directed towards the role of microglia in responding to disease and injury, here, we examine the role of microglia in the normal retina and broader visual system, including their contribution to the developing nervous system and maintaining normal retinal structure and function. In particular, we compare what has been learned from studies in the visual system with other regions of the CNS, to highlight the common functions of microglia across the normal nervous system.

## Microglial Genesis and Cns Colonization

Microglial ontogeny has historically been a hotly debated topic within the literature, with early work suggesting a neuroectoderm origin similar to other neurons/glia within the CNS. However, relatively more recent work has identified that microglia arise from embryonic yolk sac progenitors (Alliot et al., 1999). Their development and ongoing survival are dependent on several factors including the transcription factor Spi-1 Proto-Oncogene (PU.1), colony stimulating factor receptor (CSF1R) and interferon regulatory factor 8 (IRF8). Once differentiated, microglia colonize the developing brain from embryonic day (E) 8.5–9.5, while this occurs a little later in the retina (~E11.5) after invasion *via* the ciliary margin (Santos et al., 2008; Ginhoux et al., 2010). In both the brain and retina, there appears to be two waves of microglial infiltration into the respective tissues, with the second wave entering the brain and retina prior to the formation of the blood-brain-barrier (BBB) at E12.5, but after retinal vascularization (Chen et al., 2002; De et al., 2018). Once present, microglia distribute throughout the brain adopting a spatiotemporal distribution pattern dependent on local signal cues. Within the retina, microglia initially reside within the NFL and eventually adopt a bilayer distribution residing within the synaptic layers (OPL and IPL) whilst extending their processes throughout the whole tissue (Santos et al., 2008; Figure 1). As shown in Figure 1, microglia exhibit a number of morphologies and connections within the rodent, primate and human retina, with some cells contacting synapses in both plexiform layers of the retina (Figure 1B). The colonization of the retinal and brain microglial populations, early in development and prior to several key neural/vascular developmental stages, has them uniquely placed to play a role in subsequent refinement and maturation of the CNS. Additionally, as microglial processes contact neurons, glia, and blood vessels, they can provide structural support and functional refinement to most cells within the CNS.

## Microglia and The Control of Neurogenesis

Formation of the normal CNS requires careful regulation of the number and differentiation of neural progenitor cells, a process that is thought to depend on microglia. Indeed, ablating microglia with clodronate is associated with an increase in the number of neural precursor cells in the cerebral cortex (Cunningham et al., 2013). However, in a somewhat contradictory study, genetic ablation of the microglial-specific Csf1r was associated with a reduction in the number of basal progenitors in the subventricular zone (SVZ; Arno et al., 2014). This somewhat confusing result may reflect a dual role of microglia in supporting neurogenesis on the one hand and also removing progenitors on the other (Shigemoto-Mogami et al., 2014). Within the cerebellum, the phagocytic capacity of microglia was observed to be critical in the developmental loss of Purkinje cells, with microglial-induced superoxide ions playing a major role in Purkinje cell death at early postnatal ages (Marín-Teva et al., 2004).

While there are no studies investigating the role of microglia in neurogenesis within the visual centers of the brain, a possible role for microglial regulation of neurogenesis has been suggested in the retina, at least in lower vertebrates. Using the zebrafish, Huang et al. (2012) showed that morpholino knockdown of Csf1r (encoded by the zebrafish gene, *fms*) resulted in delayed macrophage/microglial infiltration of the retina, and microphthalmia. Further investigation showed Csf1r knockdown to delay neurogenesis and neural differentiation, with most major retinal cell types absent in the mutant (Huang et al., 2012). These observations have been confirmed in knockdown of the microglial specific gene Progranulin-a (Pgrn-a) in morpholinos, with retinal progenitors failing to exit the cell cycle leading to delayed neurogenesis and microphthalmia (Walsh and Hitchcock, 2017). Importantly, both these studies showed that when embryos were allowed to survive beyond the time of morpholino inhibition, retinal architecture partially recovered. In the murine retina, Csf1r inhibition or genetic ablation *via* CRISPR-CAS9 did not affect gross retinal structure, however, it did alter proliferation and survival of precursor cells (Ferrer-Martín et al., 2015; Kuse et al., 2018; Pridans et al., 2018). Additionally, microglial IL-6 prevented human retinal progenitor cells from forming neurospheres *in vitro*, further suggesting that microglia likely regulate neurogenesis in the mammalian retina (Balasubramaniam et al., 2009).

Microglia also play a role in adult neurogenesis, a more radical form of plasticity that involves the production of new neurons in the mature CNS to facilitate learning and memory formation (Deng et al., 2010; Rodríguez-Iglesias et al., 2019). Initial indirect evidence for microglial involvement was provided by studies showing adult neurogenesis was inhibited by neuroinflammation and restored by anti-inflammatory intervention (Ekdahl et al., 2003; Monje et al., 2003), while environmental enrichment induced neurogenesis, inhibited microglial activation, and inhibited neuroinflammation (Gong et al., 2018; Mee-Inta et al., 2019). Direct involvement of microglia in adult neurogenesis has also been shown with “quiescent” microglia involved in clearance of newborn cells in the hippocampus (Sierra et al., 2010). Indeed, microglial depletion in the dentate gyrus, where neuronal stem cells reside, prevents hippocampal adult neurogenesis by impairing neuroblast survival (Kreisel et al., 2019). *In vitro* studies have implicated microglial soluble factors in the regulation of adult neurogenesis (Walton et al., 2006; Matsui and Mori, 2018). Indeed, signals known to guide microglia toward dying cells including purines such as ADP acting *via* the receptor P2Y13 or fractalkine acting on its receptor Cx3cr1 have been implicated (Bolós et al., 2018; Stefani et al., 2018).

In contrast to the brain, adult neurogenesis does not appear to contribute to retinal homeostasis, with the exception of non-mammalian vertebrates such as zebrafish (Lamba et al., 2008). Interestingly, unlike the brain, retinal regeneration is dependent on the activation of microglia (Mitchell et al., 2018), with anti-inflammatory treatments impairing regeneration, while injection of the pro-inflammatory cytokine, IL-6, induces regeneration (Fischer et al., 2014; Silva et al., 2020). Providing direct evidence of the importance of microglia, ablation of microglia with clodronate prevents Müller cells from producing retinal progenitors after *N*-methyl-D-aspartate (NMDA)-induced damage (Fischer et al., 2014).

## Microglial Induction and Clearance of Apoptotic Cells

In addition to neurogenesis, microglia can contribute to CNS development by regulating the death and clearance of neurons. During embryogenesis, programmed cell death operates in tandem with neurogenesis to refine neuronal circuitry. Being the primary phagocytic cell in the CNS, microglia are responsible for the clearance of dead or dying cells. In addition, there is evidence suggesting that microglia can trigger the onset of neural cell death.

### Contribution of Microglia to Programmed Cell Death

Programmed cell death in the developing CNS is thought to occur *via* multiple mechanisms, of which, apoptosis is the most well understood (Zakeri et al., 2015). Apoptosis involves the induction of a signaling cascade that ultimately leads to break down of a cell’s proteome by activated caspases (Elmore, 2007). Induction of this process depends on the balance between pro-death and pro-survival signals, which can be regulated by glia (Lago-Baldaia et al., 2020). Microglia in particular may mediate induction of apoptosis in a number of ways depending on the developmental context. In the developing motor circuit for example, excess motor neurons undergo apoptosis *via* a mechanism involving activation of TNF receptor 1 by tumor necrosis factor-α (TNF-α), a cytokine produced by microglia (Sedel et al., 2004). In the cerebellum, microglia can induce developmental cell death of Purkinje cells by release of superoxide ions (Marín-Teva et al., 2004). Microglial release of superoxide ions can also induce cell death in the developing hippocampus, which is controlled by microglial expression of the integrin CD11β and the immunoreceptor DAP12 (Wakselman et al., 2008).

In the retina, microglia also contribute to developmental cell death (Vecino et al., 2004). In fact, some of the earliest direct evidence for the contribution of microglia to developmental cell death in the CNS came from the study of cell death in the embryonic chick retina. In this study microglial-derived nerve growth factor (NGF) was shown to induce cell death at an early age in the retinal neuroepithelium (Frade and Barde, 1998). This function was hypothesized to create space for developing retinal ganglion cell axons (Frade and Barde, 1998). More recently, studies involving microglial ablation have confirmed the importance of microglia-mediated cell death in the developing mammalian retina. In one study, depletion of microglia by loss of Csf1r resulted in decreased developmental apoptosis and increased density of retinal ganglion cells (Anderson et al., 2019). Similarly, depletion of microglia in a conditional CX3CR1-Cre^ER^-iDTR, in which microglia are selectively ablated by tamoxifen induced expression of a diphtheria toxin receptor, showed altered density and distribution of astrocytes (Puñal et al., 2019). Importantly, the reduction in astrocyte density normally observed during postnatal development was reduced in CX3CR1-Cre^ER^-iDTR, highlighting the importance of microglial phagocytosis of astrocytes in regulating astrocyte density (Puñal et al., 2019).

Programmed cell death is also important for the development of vision processing areas of the brain, such as the optic tectum, which is reported to contain more apoptotic cells than any other area in the developing zebrafish CNS (Bachstetter et al., 2011). However, while microglia coincide spatiotemporally with cell death in developing optic pathways (Martín-Partido and Navascués, 1990; Cole and Ross, 2001; Bejarano-Escobar et al., 2011), direct microglial involvement has not been demonstrated.

### Microglial Clearance of Apoptotic Cells

Following apoptosis, cell debris must be cleared from the developing CNS *via* phagocytosis. Since circulating macrophages are excluded from the CNS by the blood brain barrier, clearance of debris is primarily performed by resident microglia, although other glial cells are known to have some phagocytic capacity (Neumann et al., 2009; Galloway et al., 2019). Real-time clearance of apoptotic cells by microglia was first observed by *in vivo* imaging of the embryonic zebrafish brain (Mazaheri et al., 2014). Microglia were seen to extend processes that reached out and formed phagosomes around apoptotic cells. The exact mechanisms by which microglia detect and phagocytose apoptotic cells are not well understood but may involve the expression of so-called “eat me” signals that are recognized by phagocytic cells. One of these signals has been identified as phosphatidylserine (PS), which is exposed on the surface of dying cells and microglial PS receptors MFG-E8, BAI1 and TIM-4 have been shown to facilitate the phagocytosis of apoptotic cells (Hanayama et al., 2002; Liu et al., 2013; Mazaheri et al., 2014). In addition to cell surface “eat me” signals, clearance of apoptotic cells requires longer range signaling from chemotactic factors that attract microglia. Signals that have been identified to guide microglia toward apoptotic cells including ATP or ADP acting *via* microglial P2Y12 receptors, and fractalkine, acting *via* microglial CX3CR1 (Sieger et al., 2012; Sokolowski et al., 2014). Interestingly, microglia have also been observed phagocytosing viable neural precursor cells and oligodendrocyte precursor cells, inducing a type of cell death known as “phagoptosis” (Cunningham et al., 2013; Nemes-Baran et al., 2020).

Clearance of cell debris in the retina is also thought to be primarily achieved *via* phagocytosis by microglia, although Müller cells have also been shown to exhibit phagocytic activity (Bejarano-Escobar et al., 2017). Early work in aldehyde-fixed tissue localized microglia to areas of cell death in the developing retina, providing the first evidence for microglial involvement in the clearance of dying retinal neurons (Hume et al., 1983). This was supported by later work that showed the appearance of phagocytic microglia coincided with peak ganglion cell death in the postnatal retina (Bodeutsch and Thanos, 2000). Several possible receptors have been suggested to regulate microglial phagocytosis in the retina, including toll like receptors, Dectin-1, PS receptors, MerTK, and TREM2 (Maneu et al., 2011; Kochan et al., 2012; Li, 2012, 2013). However, these receptors have only been implicated in clearance of dying cells during retinal degeneration, and not during retinal development. A recent study revealed phagocytosis during retinal development is mediated by microglial P2RY12 (Blume et al., 2020). Using real-time imaging, the authors showed that inhibiting P2RY12 signaling resulted in greater numbers of apoptotic cells. Rather than increased levels of cell death, this was due to delayed clearance of apoptotic cells by microglia.

While the exact mechanisms mediating cell death and clearance during CNS development are yet to be fully determined, microglia are likely to play an important part. As the primary phagocytic cell, they are especially qualified to refine the neural population by removing entire cells. Similarly, they are also suited for more specific fine-tuning of neural circuitry by mediating the formation and removal of individual synapses, which is crucial for postnatal maturation of neurons.

## Microglial Involvement in Neuronal Refinement

During embryogenesis and early post-natal development, the neuronal components of the CNS undergo maturation and refinement. This refinement is dependent on the type of neuron as well as the specific environment. Work within the last decade or so has identified microglia to have a role in a number of these processes.

### Neuronal Maturation

**Figure 3:**
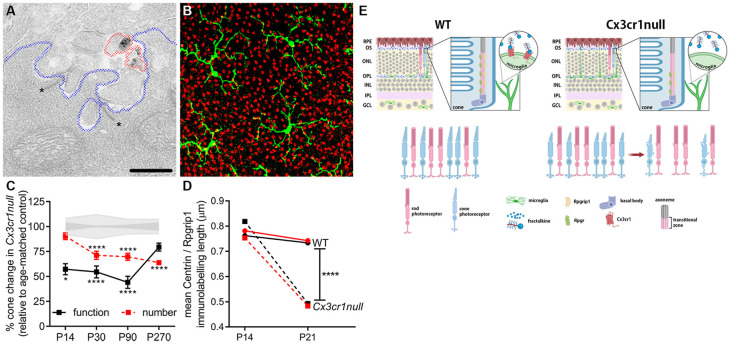
Microglia are important in the post-natal maturation of cone photoreceptors. **(A)** Microglia make contact with photoreceptor synapses as shown in the electron micrograph, with Cx3cr1-EGFP-labeled microglial processes (black deposit, circled in red) in close proximity to photoreceptor ribbons (asterisk, *), within the synapse (outlined in blue). **(B)** Microglia (green; Iba-1) are also observed to contact multiple cone photoreceptors (red; peanut agglutinin, PNA) within the outer retina. **(C)** During cone photoreceptor postnatal development, function is reduced in Cx3cr1null animals from around P14 (electroretinogram, black squares), while cones are lost from 1 month of age (PNA quantification, red squares). *p < 0.05, ****p < 0.0001. **(D)** During outer segment elongation which occurs after eye opening (>P14) there is a reduced length of centrin (black squares) and Rpgrip1 (red squares) expression within the cone photoreceptor cilium of the Cx3cr1null mice compared to wildtypes (circles). **(E)** A schematic showing that microglial communication with cone photoreceptors *via* the receptor Cx3cr1 is important in postnatal maturation, with loss of this receptor (Cx3cr1null) leading to restricted expression of key cilium proteins during eye opening, reduced outer segment elongation, dysfunction and ultimately cell death (Jobling et al., 2018b).

At present, most neuronal maturation described within the CNS involves refinement of synapses (see below). However, the light detecting photoreceptors within the retina exhibit a unique activity-dependent maturation, as well as unique contact between cone photoreceptor synapses and microglial processes (Figure 3). The contact between microglia processes and a cone photoreceptor terminal is shown in Figure 3 at the ultrastructural level as well as using high resolution confocal microscopy. Importantly, after eye opening (>P14 in mouse) both rod and cone photoreceptors elongate their outer segments and increase expression of their respective photopigments, in order to maximize their functional output (Timmers et al., 1999). This process is dependent on protein transportation *via* a specialized form of a primary cilium, which is located between the inner and outer segments (Steinberg et al., 1980). Using the Cx3cr1-EGFP knock-in mouse (Jung et al., 2000), we showed that cone maturation was aberrant when this microglial-specific receptor was genetically ablated and that this led to early cone photoreceptor death (Jobling et al., 2018b; summarized in Figure 3E). Specifically, during eye opening, the loss of microglial Cx3cr1 resulted in aberrant expression of the cilium-related genes *Rpgr* and *Rpgrip1*, altered protein localization within the cilium and a failure to exhibit an increase in opsin expression. These changes resulted in cone photoreceptors with shortened outer segments and reduced function, which ultimately resulted in cone photoreceptor loss by P30 (Figure 3; Jobling et al., 2018b). While there is indirect evidence supporting a role for microglia in the maintenance of ciliated dendritic endings in olfactory sensory neurons *via* galectin-3 (Comte et al., 2011), this microglial regulation of the photoreceptor cilium appears to be retina-specific.

In addition to microglial-controlled photoreceptor maturation, Burger et al. (2020) showed that microglia are likely also involved in controlling neurite growth within the outer retina. Their work showed that microglial C1q expression was critical in confining retinal horizontal cell neurites to the outer retinal synaptic layer (OPL), with C1q knock-out mice exhibiting neurite extensions into the outer retina at the time of eye opening. Interestingly, this effect was specific to horizontal cells, with no evidence of similar differences in cone photoreceptors or bipolar cells (Burger et al., 2020). This regional specificity in neuronal refinement is similar to that seen in the brain where C1q is needed in the lateral geniculate nucleus (LGN), which is not critical in the visual cortex (Stevens et al., 2007; Welsh et al., 2020).

### Synaptogenesis and Synapse Refinement

As microglial invasion of the developing brain and retina precedes the presence of other support cells such as astrocytes, oligodendrocytes and retinal Müller cells (Ginhoux et al., 2010; Kettenmann et al., 2013), it is likely that microglia play a role in embryonic synaptogenesis and synaptic refinement. Despite this, most work investigating microglial involvement is limited to early postnatal ages where synaptogenesis is coincident with a dramatic increase in the density of cortical microglia that reaches a maximum by P18 (Dalmau et al., 1998, 2003). This indirect association of microglial involvement in maturation of the CNS was confirmed by Miyamoto et al. (2016) who demonstrated that direct microglial contact with pyramidal neurons in the somatosensory cortex induced filopodia formation on dendritic spines (Weinhard et al., 2018). In addition, genetic ablation of microglia resulted in decreased spine density, functional excitatory synapses and reduced connectivity. Despite these data, there are no studies in the retina or higher order vision processing centers within the brain that have examined microglial control of synaptogenesis. However, single cell transcriptomics of cells in the LGN suggest microglia up-regulate the synaptogenic gene, Hevin, early in postnatal development at a time co-incident with the period of increased synaptogenesis (Kalish et al., 2018).

In addition to the formation of new synapses, microglia are also required to fine tune neuronal circuits by selectively removing non-functional synapses, as well as refinement of established synapses (Paolicelli et al., 2011). Some of the earliest indications of microglial involvement in so called “synaptic pruning” (Blinzinger and Kreutzberg, 1968) were based on the observation that microglia were enriched in areas undergoing active synaptic remodeling (Dalmau et al., 1998), and the idea that microglial-synapse contact was dependent on neuronal activity (Wake et al., 2009; Tremblay et al., 2010). In fact, within the mouse primary visual cortex (V1), microglia normally show a preference for smaller and transiently growing dendritic spines, however, with altered light exposure or visual deprivation, microglia underwent morphological change, showed the presence of phagocytic structures, and exhibited altered synapse contact (Tremblay et al., 2010). This activity dependent effect was also observed in the mature mouse cortex (Wake et al., 2009).

Using the LGN as a model system, Schafer et al. (2012) explored the role of microglia in the elimination of ganglion cell inputs during early postnatal development. Their work showed that during postnatal synaptic remodeling (P5 in mouse) microglial processes and lysosomes contained presynaptic inputs. Reflecting the work in the visual cortex, Schafer et al. (2012) also found that this microglial dependent engulfment was activity dependent. Indeed, they showed that reduced neural activity following tetrodotoxin (TTX) treatment was associated with an increase in microglial elimination of ganglions cell inputs, while forskolin-dependent increase in neural activity lead to a decrease in pruning (Schafer et al., 2012). Rather than this elimination simply reflecting the ability of microglia to phagocytose already “pruned” synapses, microglia actively engulf synapses. Specifically, complement proteins C3 and C1q expressed by neurons activate complement receptor CR3 on microglia to trigger elimination of synapses (Stevens et al., 2007; Schafer et al., 2012), while neuronal CD47 signals to microglial SIRPα to prevent pruning (Lehrman et al., 2018). However, deletion of C1q or C3 only impaired the development of synaptic connections rather than completely abolishing it, suggesting the involvement of additional pathways in synaptic pruning (Stevens et al., 2007; Schafer et al., 2012). One such possible pathway involves the microglial receptor Cx3cr1, with loss of Cx3cr1 signaling impairing the development of glutamatergic synapses in the brain, causing abnormal hippocampus structure (Paolicelli et al., 2011; Hoshiko et al., 2012). However, this was not replicated in the visual cortex, where ablation of Cx3cr1 did not affect synaptic pruning (Lowery et al., 2017; Schecter et al., 2017). Similarly, TGF-β and serotonin have also been shown to modulate microglial involvement in synaptic refinement (Bialas and Stevens, 2013; Kolodziejczak et al., 2015).

Within the retina, the role of microglia in control of early postnatal synapse refinement is yet to be fully explored. Indirect evidence suggests a possible involvement, with activity dependent synapse formation and microglial change evident within the retina, while genetic ablation of microglia in the adult mouse resulted in a progressive decline in cone-mediated function and degeneration of photoreceptor synapses (Fontainhas et al., 2011; Wang et al., 2016). Supporting an ongoing role for microglia in maintenance of neuronal function in the adult retina, mice deficient in IL-34, a ligand for CSF1R, exhibited a specific loss of microglia in the IPL and associated impairment of bipolar cell function (O’Koren et al., 2019). The exact signals released by microglia that are necessary for this maintenance are currently unknown, but likely candidates are thought to be neurotrophic factors, which are known to mediate many important aspects of CNS homeostasis.

## Microglial Trophic Support

An important component of intercellular communication is signaling *via* neurotrophic factors, a family of cytokines that have long been known to contribute to the development and maintenance of the CNS (Henderson, 1996). Broadly, these factors promote the growth, survival, and differentiation of neurons and glia and can be protective in models of neurodegeneration (Fumagalli et al., 2008). Increasing evidence suggests that microglial secreted factors play a role in the maintenance and support of cells within the CNS, including visual system.

Neurotrophic factors and their associated receptors are widely expressed by cells within the CNS, including microglia. Microglia are known to secrete a range of neurotrophic factors that can facilitate some of the many functions these cells perform within the developing and mature CNS (Elkabes et al., 1996; Nakajima et al., 2001; Hanisch, 2002). In fact, it has been suggested that microglia may constitutively express neurotrophic factors for CNS homeostasis, and that the developmental deficits caused by inhibition of microglial CSF1R could be a result of reduced expression of such factors (Hanisch, 2002; Erblich et al., 2011). As an example, microglia-derived insulin like growth factor I (IGF-1) is necessary for postnatal survival of Layer V cortical neurons (Ueno et al., 2013), and for the development and maintenance of oligodendrocytes (Hagemeyer et al., 2017). In the mature brain, microglial IGF-1 is thought to underlie the neurogenic effect of exercise (Kohman et al., 2012), while microglial brain derived neurotrophic factor (BDNF) is required for long term and spatial memory formation (Parkhurst et al., 2013). Further, microglia-derived neurotrophic factors likely play an essential role in brain injury and disease, as upregulation of microglial IGF-1 is associated with improved outcomes in models of ischemia, Alzheimer’s disease, and amyotrophic lateral sclerosis (O’Donnell et al., 2002; Butovsky et al., 2006; Lalancette-Hébert et al., 2007; Chiu et al., 2013).

While the above examples illustrate the importance of microglial neurotrophic signaling in brain development, plasticity, and disease, the significance of microglial neurotrophic signaling in retinal development and homeostasis is less clear. Work in the chick embryo has detailed the importance of microglial nerve growth factor (NGF) in the programmed cell death of retinal neurons during embryogenesis (Frade and Barde, 1998). In this work, microglia were observed to be the sole source of NGF in the E4–5 chick retina, while removal of microglia inhibited cell death likely *via* the neurotrophin receptor p75. Other neurotrophic factors that can be expressed by microglia, such as ciliary neurotrophic factor (CNTF), leukemia inhibitory factor (LIF), IGF-1, and fibroblast growth factors (FGFs), are known to contribute to retinal development and homeostasis, but microglia have not been confirmed as the source of these factors in development (Otteson et al., 2002; Martinez-Morales et al., 2005; Rhee and Yang, 2010). Despite the lack of direct evidence for microglial neurotrophic support in the normal retina, there has been a body of work exploring this during disease. Most notably, work by Harada et al. (2002) showed microglia to indirectly promote cell survival during retinal degeneration by communicating with Müller cells *via* neurotrophic factors such as nerve growth factor (NGF), CNTF, and BDNF. Additionally, IGF-1 has been shown to protect against photoreceptor and RGC degeneration in a model of retinitis pigmentosa (Arroba et al., 2011), with the positive effects diminished in the absence of microglia.

While several studies suggest that activation of retinal microglia is neuroprotective (Bruban et al., 2011; Fontainhas et al., 2011; Ferrer-Martín et al., 2015), excessive microglial activation can cause retinal degeneration and impair their ability to mediate neural plasticity (Roque et al., 1999; Ekdahl et al., 2003; Yang et al., 2007; Costello et al., 2011). Therefore, proper tissue homeostasis depends on inhibiting microglia activation and maintaining their neuro-protective state. This is achieved by immunomodulatory cytokines that are constitutively expressed by retinal pigment epithelial (RPE) cells, neurons, vascular endothelial cells, and Müller cells (Langmann, 2007). For example, TGFβ released by RPE cells triggers the production of the anti-inflammatory cytokine IL-10 by microglia, which results in down-regulation of antigen presenting proteins MHC-II, CD80, and CD86 (D’orazio and Niederkorn, 1998). Similarly, retinal neurons and vascular endothelial cells express the transmembrane protein CD200, which inhibits activation upon binding to microglial CD200R (Broderick et al., 2002), while retinal neurons also express the chemokine fractalkine, which binds to microglial CX3CR1 to prevent microglial activation (Liang et al., 2009). Additionally, CD200 and fractalkine signaling are reported to mediate microglial motility and migration within the healthy retina (Carter and Dick, 2004; Liang et al., 2009). Finally, Müller cells can reverse microglial activation by producing diazepam binding inhibitor (DBI) which binds to translocator protein (TSPO) expressed by activated microglia (Wang et al., 2014). Taken altogether, these findings illustrate that microglia may provide crucial trophic support to the developing and mature visual system, which is facilitated by the bi-directional communication between microglia and several other cell types.

## Microglia and Glial Function

Within the CNS, glial cells such as astrocytes, oligodendrocytes and the retinal-specific Müller cells perform critical roles in development and homeostasis. Generally, the development and regulation of these cells have received less attention compared to their neuronal counterparts and therefore there is a distinct lack of detail regarding microglial-glial interaction and how these two cell types impact on each other’s function. Generally, most interest has been directed at the bi-directional communication during injury and disease (Conedera et al., 2019; De Waard and Bugiani, 2020). Despite this there is evidence of a microglial role in glial development within the brain and retina and increasing interest in how these two cell types communicate to maintain normal tissue homeostasis.

Work in the neural stem cell rich SVZ of the forebrain early in post-natal development (P2–P4) has indicated that microglia are important for oligodendrogenesis, with minocycline-inhibition of microglial activation resulting in decreased numbers of oligodendrocyte progenitors and mature oligodendrocytes (Shigemoto-Mogami et al., 2014). This effect was observed to be dependent on cytokines such as IL-1β and IL-6. Depletion of microglia has also been shown to reduce numbers of NG2+ oligodendrocyte precursor cells and subsequent myelination in the corpus callosum and cerebellum, while a similar disruption of oligodendrocyte precursor cell maturation and migration occurs in the hypothalamus after PLX5622 (CSF1R blockade) ablation (Hagemeyer et al., 2017; Marsters et al., 2020). Interestingly, this effect may not be restricted to early post-natal development, as oligodendrocyte precursor cell homeostasis in the adult brain is also dependent on microglia (Hagemeyer et al., 2017).

Single cell transcriptome analysis within the LGN supports a role of microglia in myelination, with extensive gene expressional change occurring during eye opening (P10–P16). During this period of change, Kalish et al. (2018) found microglia to significantly increase their expression of the key myelination gene, Autotaxin (*Atx*). Autotaxin is responsible for the production of lysophosphatidic acid (LPA) which binds to LPA receptor 1 (LPAR1) in oligodendrocytes to facilitate myelination. Coordinated with this microglial *Atx* increase, oligodendrocytes increased LPAR1 expression, highlighting a possible pathway for microglia to regulate myelination within the LGN at a time when visual experience requires significant remodeling.

Within the retina, microglial involvement appears to be important for the developmental reduction in astrocytes. Relatively recent work has detailed significant early post-natal reduction (3-fold) in astrocyte numbers between P5 and P14 within the mouse retina that was independent of classical apoptosis (Puñal et al., 2019). Specific depletion of microglia using the Cx3cr1-creER-iDTR system resulted in increased astrocyte numbers, aberrant astrocyte morphology and subsequent retinal vascular pathology. The overall mechanism was dependent on non-apoptotic microglial phagocytosis, however, this developmental astrocyte reduction was not fully blocked when microglia were ablated due to astrocyte-dependent phagocytosis (Puñal et al., 2019). Other work has also shown the presence of novel glial cells within the chick retina [non-astrocytic inner retinal glial-like (NIRG) cells] to rely on microglia, with clodronate ablation leading to a 95% loss of NIRG cells over 7 days (Zelinka et al., 2012). With respect to Müller cells, there is no evidence of a similar developmental role, however microglial ablation has been reported to reduce the formation of Müller cells progenitor cells in avian and zebrafish models (Fischer et al., 2014; Conedera et al., 2019). Apart from a developmental role, microglial-glial communication is known to occur in order to maintain normal tissue homeostasis (Jha et al., 2019), however, most of this is based on *in vitro* experiments or inferred from anatomical contacts. In the context of neuromodulation, both microglia and Müller cells respond to changes in retinal neuronal activity, however, work suggests that microglia rely on indirect activation *via* ATP to respond to changes in neuronal activity rather than direct detection of neurotransmitters (Fontainhas et al., 2011; Li et al., 2012). It has been suggested that Müller cells provide this ATP signal in response to neuronal activity, suggesting microglial-Müller cell communication is an ongoing mechanism responsible for the maintenance of retinal homeostasis (Wang and Wong, 2014).

## Microglia and Vasculature

The CNS contains the most metabolically active organs in the body, with endogenous neurons dying within just a few minutes of oxygen deprivation (Richmond, 1997). Reflecting this, the brain demands 20% of the body’s energy supply despite constituting only 2% of body weight (Zhu et al., 2009; Magistretti and Allaman, 2015). The retina is one of the most energy dependent systems within the brain, despite having very little capacity for energy storage (Kooragayala et al., 2015). The CNS therefore requires an efficient and tightly controlled blood supply, that can rapidly respond to changes in metabolic demand. While astrocytes and Müller cells have been shown to be the major regulators of vascular growth and modification, microglia are known to also contribute to normal vascular development and recent preliminary work also identifies a role in vascular regulation in the CNS.

### Vascular Development

Microglia begin to populate the brain and the retina prior to the development of vasculature (Cuadros et al., 1993; Rymo et al., 2011). Numerous studies in the brain and retina have shown close approximation of microglia with developing vessel tips (Ashwell et al., 1989; Checchin et al., 2006). In fact, studies in the cortex have shown that endothelial cells extend processes that directly contact both neural precursor cells and microglia (Penna et al., 2020). Providing direct evidence of a functional role, loss of microglia in PU.1 mutant mice resulted in reduced blood vessel intersections in the hindbrain (Fantin et al., 2010). Similarly, CSF1R blockade (PLX5622), which depletes macrophages including microglia, resulted in mouse choroidal vascular atrophy, RPE disorganization and dysfunction, as well as altered angiogenic growth factor expression (Yang et al., 2020). *In vitro* evidence suggests that the microglial regulation of blood vessel growth may occur *via* the increased expression of ephrin-A3 and -A4 (Li et al., 2014).

Within the retina, studies have also shown a close relationship between microglia and blood vessels, with microglia contacting endothelial tip cell filopodia which are thought to guide vessel growth (Checchin et al., 2006). Studies that use pharmacological or genetic ablation of microglia provide compelling evidence for a direct role for microglia in retinal vessel formation. Checchin et al. (2006) showed that clodronate liposomal depletion of microglia reduced retinal vascular development in the mouse, while this was restored following intraocular injection of microglia. Similarly, Kubota et al. (2009) showed that genetic ablation of Macrophage colony-stimulating factor (M-CSF, in Csf1op/op mice) impaired early postnatal development of retinal vasculature (P2–P4) with reduced branching and altered arterio-venous patterning. This vascular defect was independent of VEGF and resolved by 3 weeks of age, suggesting that microglia may not be important for adult maintenance of the retinal vasculature. This apparent restoration of the vasculature was explained in later work by Fantin et al. (2010) that showed the resolution of the vascular phenotype to be due to reduced vessel pruning at later stages.

With respect to the exact mechanism of this microglial mediation of vascular growth, the chemokine receptor CX3CR1, which is primarily expressed by microglia in the healthy CNS, has been shown to mediate endothelial cell migration and tube formation in cell culture (Volin et al., 2001). A more recent study has shown that genetic deletion of the Angiotensin (1–7) receptor MAS (Mas1^−/−^), resulted in impaired retinal vascular development due to reduced microglial number at the developing vascular front (Foulquier et al., 2019). Interestingly, the activation of MAS upregulates the Notch signaling pathway, which has been linked to microglial localization and interaction with endothelial cells within the retina (Foulquier et al., 2019; Haupt et al., 2019).

### Blood-Brain and Blood-Retinal Barrier and Neurovascular Unit (NVU)

The presence of the blood-brain and blood-retinal barriers (BBB and BRB, respectively) are critical for providing a physical and biochemical separation between the CNS and peripheral circulation, thereby establishing the unique microenvironment that ensures proper neuronal function. While most work has concentrated on the role of endothelial cells, astrocytes and pericytes in the formation and maintenance of the BBB and BRB (Cheslow and Alvarez, 2016), the close proximity between microglia and blood vessels makes them ideally placed to contribute (Ronaldson and Davis, 2020). *In vitro* co-culture experiments support such a role, with brain-derived endothelial cells increasing their expression of the tight junction protein occludin when co-cultured with unstimulated microglia (Mehrabadi et al., 2017), while microglial exposure increased retinal microvascular endothelial cell proliferation (Ding et al., 2018). Furthermore, single cell RNAseq analysis of microglial subpopulations within the brain shows expression of the critical barrier gene, Claudin-5 (Li et al., 2019). Despite these indications, direct evidence for a role of resident microglia in the formation and physiological maintenance of the respective barriers remains unsupported. Studies in which microglia have been depleted show no alteration in barrier permeability within the brain, spinal cord or retina (Kokona et al., 2018; Halder and Milner, 2019; Haruwaka et al., 2019). It is therefore likely microglia do not play a significant role in the formation of the BBB and BRB, however more detailed work is required to identify possible minor contributions.

Despite providing separation from the peripheral circulation, the blood-brain and blood-retinal barriers are not static structures and have dynamic boundaries that require regulation in order to adequately supply the energy needs of neurons. This regulation is achieved through a coordinated intercellular communication *via* the neurovascular unit (NVU), encompassing neurons, glia (astrocytes and Müller cells), microglia, pericytes and endothelial cells. Such a coordinated response enables the neuronal energy requirements to be met through alterations in vascular response (neuro-vascular coupling). At present, most work investigating a microglial role in the NVU has been limited to injury and/or pathology, with activated microglia increasing permeability in the brain, spinal cord and retina, whilst also decreasing occludin, ZO1 and claudin-5 expression (Kokona et al., 2018; Halder and Milner, 2019; Haruwaka et al., 2019).

**Figure 4:**
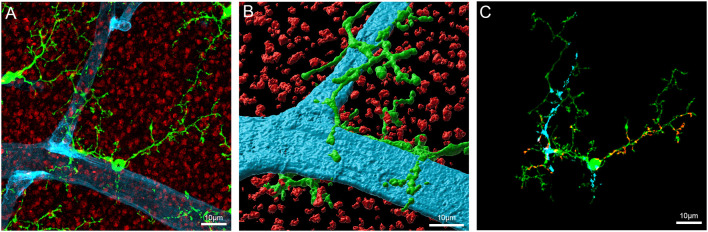
Microglia contact components of the retinal vasculature. **(A)** Flatmount of the Cx3CR1^+/GFP^ mouse retina imaged at the level of the outer plexiform layer labeled for the presynaptic terminal marker, VGLUT1 (red), and the blood vessel marker, IB4 (blue). GFP labeled microglia are visible as green cells abutting the blood vessels. **(B)** Imaris rendered microglia abutting the blood vessel in panel **(A)**. **(C)** Image showing areas of contact between blood vessels, synapses and the indicated (*) microglia in panel **(A)**. Putative contacts between microglia and blood vessels or synapses were defined by the apparent colocalization of fluorescence. The blue shading indicates areas of contact between the microglia and blood vessel, the red shows areas of contact between microglia and synapses.

As shown in Figure 4, microglial processes wrap around retinal capillaries and can also contact neural synapses, suggesting a possible role in local blood vessel control. Despite little direct evidence for an active role for microglia in the NVU in the healthy brain or retina, several studies have identified factors that could enable microglia to play a role in vascular regulation. Work in our laboratory has identified the presence of the vasoactive agent angiotensinogen in isolated retinal microglia (Jobling et al., 2018a), complementing previous studies identifying components of the renin-angiotensin system (RAS) within microglia (Liu et al., 2018; Phipps et al., 2018). It is well known that the RAS can regulate CNS vessels and preliminary work in our laboratory suggests that microglia are capable of regulating retinal capillary diameter (Jobling et al., 2018a; Dixon et al., 2019). While further work is required to fully document this novel role for microglia, it may represent an additional vasoregulatory pathway to the Müller cell dependent pathway described within the retina (Metea and Newman, 2007).

## Conclusion

Since their first identification in the CNS, microglia have been heavily studied for their contribution in injury and disease. However, over the last decade the importance of microglia in maintaining normal structure and function of the nervous system has emerged. High resolution *in vivo* imaging and selective ablation methods have enabled researchers to identify a role for these cells in normal development and maturation. Most of these are based on the dynamic nature of microglia and the fact that the resident population is established within the CNS relatively early in development and maintained throughout the life of the organism. A number of these roles have been described within the retina and higher visual centers due to the relative ease of imaging and the ability to modify light-dependant maturation. Indeed, it is now clear that microglia regulate the number of neurons present within the retina and brain during development, refine synaptic connections during remodeling periods and contribute to maturation of neural circuits. In addition, microglia appear to be important in regulating the function of the vasculature. While initially described as the resident immune cell within the CNS, undertaking macrophage-like functions, microglia are now becoming known for their critical roles in establishing and maintaining the normal tissue architecture within CNS and visual system.

## Author Contributions

MD wrote parts of the initial draft and created some of the figures. UG edited drafts and created a figure. EF and AJ wrote part of the initial draft, edited the final draft and created a figure. All authors contributed to the article and approved the submitted version.

## Conflict of Interest

The authors declare that the research was conducted in the absence of any commercial or financial relationships that could be construed as a potential conflict of interest.
